# Klebsiella vaccine research and development in Africa: a rapid scoping review of current evidence and future priorities

**DOI:** 10.1136/bmjph-2025-004451

**Published:** 2026-07-13

**Authors:** Chinwe Iwu-Jaja, Akhona Victress Mazingisa, Anelisa Jaca, Chidozie D Iwu, Charles Shey Wiysonge

**Affiliations:** 1 World Health Organization Regional Office for Africa, Brazzaville, Congo; 2 Department of Community Health Studies, Durban University of Technology, Durban, South Africa; 3 South African Medical Research Council, Cape Town, South Africa; 4 School of Health Systems and Public Health, University of Pretoria, Pretoria, South Africa; 5 Cochrane South Africa, South African Medical Research Council, Cape Town, South Africa

**Keywords:** Vaccination, Public Health, Epidemiology, Systematic Review

## Abstract

**Introduction:**

*Klebsiella pneumoniae* is a major cause of neonatal sepsis in Africa and a WHO priority pathogen for vaccine development. With growing antimicrobial resistance and limited treatment options, maternal vaccination offers a potential preventive strategy. This scoping review synthesises evidence on *K. pneumoniae* vaccine research in Africa and highlights gaps to guide future research and development priorities

**Methods:**

A systematic PubMed search was conducted on 1 September 2025 to identify studies on *K. pneumoniae* vaccine research in Africa. Additional searches were conducted in ClinicalTrials.gov, the Pan-African Clinical Trials Registry and the WHO International Clinical Trials Registry Platform. Eligible studies were screened, and data were extracted and synthesised using a framework focused on disease burden, vaccine development and implementation considerations.

**Results:**

A total of 14 studies published between 2008 and 2024 met the inclusion criteria, with most (11/14) published in the last 5 years. Studies reported a high burden of *K. pneumoniae* infections across multiple African countries, particularly in neonatal sepsis and meningitis. While genomic and immunological studies provided insights into vaccine target selection, no *K. pneumoniae* vaccine trials were identified in Africa. Modelling studies suggest that maternal immunisation with a 70% effective vaccine could prevent approximately 400 000 neonatal sepsis cases and 80 000 deaths annually worldwide, including Africa. Key research gaps include limited epidemiological surveillance and absence of immune correlates of protection.

**Conclusions:**

Despite the high burden of *K. pneumoniae* neonatal infections in Africa, vaccine research remains in its early stages, with no ongoing clinical trials on the continent. Strengthening surveillance systems, advancing vaccine candidates and integrating *K. pneumoniae* vaccination into existing maternal immunisation programmes are critical next steps. Multisectoral collaboration among researchers, policymakers and continental and global health organisations will be essential to accelerate vaccine development and ensure equitable access in Africa.

**WHAT IS ALREADY KNOWN ON THIS TOPIC**

- *Klebsiella pneumoniae* is a significant contributor to neonatal mortality in Africa, responsible for 21% of child deaths with high antimicrobial resistance rates (84% to ceftriaxone and 75% to gentamicin), underscoring the urgent need for preventive strategies.

**WHAT THIS STUDY ADDS**

- Despite the substantial disease burden, vaccine development faces critical challenges including the absence of immune correlates of protection, limited surveillance infrastructure and no clinical trials in African settings.

**HOW THIS STUDY MIGHT AFFECT RESEARCH, PRACTICE OR POLICY**

- Accelerating *K. pneumoniae* vaccine development for Africa requires coordinated action across stakeholders, including strengthening surveillance systems, conducting regional clinical trials and addressing implementation barriers in maternal immunisation programmes.

## Introduction

*Klebsiella* species, comprising gram-negative, encapsulated bacteria of the Enterobacteriaceae family, are widespread in the environment and frequently found in soil, water and various other surfaces.^1^ The *Klebsiella* genus includes several species, such as *Klebsiella ozaenae*, *Klebsiella rhinoscleromatis*, *Klebsiella oxytoca* and *Klebsiella pneumoniae*. Among these, *K. pneumoniae* stands out as a major opportunistic and healthcare-associated pathogen, posing significant clinical challenges. In humans, *Klebsiella* species typically reside in the nasal passages and gastrointestinal tract without causing any noticeable symptoms. However, these bacteria can cause infections when the host’s immune system is weakened, affecting individuals with conditions like diabetes, those undergoing immune suppressive therapy and patients who have received organ transplants.^2^ The pathogen primarily causes pneumonia and sepsis, with particularly severe manifestations in hospitalised and immunocompromised patients. Its clinical impact extends to meningitis and urinary tract infections, while hypervirulent strains can cause invasive diseases, including liver and lung abscesses, even in healthy individuals.^3^,^5^ While *K. pneumoniae* is predominantly associated with healthcare settings, community-acquired infections are also common. The prevalence of community-acquired pneumonia and bacteraemia shows geographical variation, with higher rates in Asian and African regions compared with high-income countries.^5^

*K. pneumoniae* has emerged as a leading cause of neonatal sepsis in low- and middle-income countries (LMICs).^6^,^10^ A meta-analysis of studies spanning 1980–2018 revealed that *K. pneumoniae* was responsible for 21% of culture-confirmed bacteraemia or sepsis cases among 84 534 neonates across 26 sub-Saharan African countries.^6^ The economic burden of neonatal sepsis and its complications in sub-Saharan Africa is substantial, estimated at US$469 billion annually. This includes both direct healthcare costs and indirect costs through lost productivity and premature mortality.^11^

The significance of *K. pneumoniae* as a major pathogen of public health importance was formally recognised in 2008 with its inclusion in the ESKAPE (*Enterococcus faecium*, *Staphylococcus aureus*, *K. pneumoniae*, *Acinetobacter baumannii*, *Pseudomonas aeruginosa* and *Enterobacter* spp) pathogens group—a group characterised by extensive antibiotic resistance.^12 13^ The global impact of antimicrobial resistance is substantial, with *K. pneumoniae* contributing to 17.5% of AMR-associated deaths and 19.9% of deaths directly attributable to AMR.^14 15^ In the WHO African region, *K. pneumoniae* is considered one of the most resistant pathogens.^16^

Studies have documented concerning resistance patterns, with one study reporting 84% resistance to ceftriaxone and 75% to gentamicin,^17^ while another found 34% of gram-negative isolates resistant to third-generation cephalosporins.^18^ Genomic studies have revealed distinct patterns between invasive and colonising strains, notably with hypermucoidy genes being more prevalent in invasive isolates,^19^ further complicating treatment approaches.

Given this substantial disease burden and growing AMR threat, *K. pneumoniae* has been identified as one of the top 10 priority pathogens for vaccine research and development in the WHO African region.^20^ There has been an established vaccination use case which targets neonates and infants through maternal immunisation, supported by a comprehensive vaccine value profile (VVP). This VVP has examined the potential benefits of maternal immunisation in protecting infants from birth through early infancy, along with vaccination strategies for other vulnerable groups.^21^ Additionally, modelling studies suggest that maternal immunisation against *K. pneumoniae* with 70% efficacy could prevent approximately 400 000 cases (95% CI 334 523 to 485 442) of neonatal sepsis and 80 000 deaths (95% CI 18 084 to 189 040) annually worldwide.

This review therefore synthesises current evidence and identifies research gaps to inform *K. pneumoniae* vaccine development priorities in the African region.

## Methods

We conducted a rapid scoping review following the Joanna Briggs Institute (JBI) methodology^22 23^ to identify *K. pneumoniae* vaccine-related research in Africa. This review followed the Preferred Reporting Items for Systematic Reviews and Meta-Analyses extension for Scoping Reviews (PRISMA-ScR) guidelines.^24^ A scoping review methodology was adopted because *K. pneumoniae* vaccine research in Africa is an emerging field with heterogeneous study designs, making it more suitable for evidence mapping and gap identification than for systematic review with meta-analysis.^24^

### Eligibility criteria

Studies were included if they: (1) addressed any aspect of *K. pneumoniae* vaccine research, including disease burden, vaccine development, immunological studies or implementation considerations; (2) were conducted in or included data from African countries; (3) were of any study design (observational, experimental, genomic, modelling); and (4) were published in English or French, with no restriction on publication year. Studies meeting the above inclusion criteria were subsequently excluded if they: (1) were conference abstracts without full-text availability; (2) reported on *K. pneumoniae* but without relevance to vaccine research or development (eg, purely clinical or therapeutic studies); or (3) were editorials, commentaries or opinion pieces without original data.

### Search strategy

We searched PubMed on 1 September 2025. PubMed was selected as the primary database for its comprehensive indexing of biomedical and public health literature, using a comprehensive search strategy (online supplemental appendix table 1). Additionally, we searched clinical trial registries including ClinicalTrials.gov, the Pan-African Clinical Trials Registry (PACTR)^25^ and the WHO’s International Clinical Trials Registry Platform (ICTRP)^26^ and ClinicalTrials.gov^27^ for completed and ongoing *K. pneumoniae* vaccine trials. PACTR was specifically included to ensure representation of clinical research conducted in African settings.

### Study selection

Retrieved articles were imported into Rayyan, a web application for conducting systematic reviews,^28^ and duplicates were removed. Two authors (CI-J and AVM) independently screened titles and abstracts against the eligibility criteria. Records deemed potentially relevant were retrieved in full text and independently assessed for eligibility by the same two authors. Disagreements at both screening stages were resolved through discussion and consensus.

### Data extraction and synthesis

Data were extracted using a standardised Excel spreadsheet, capturing author names, publication year, study country and key findings. Evidence gaps were identified based on authors’ recommendations and findings from similar studies in LMICs.

The evidence synthesis followed a strategic framework for vaccine and immunisation research that organises findings into three thematic areas: ‘assessment of disease burden and public health and economic impact of vaccines’, ‘vaccine and immunisation technology development’ and ‘implementation research’.^29^

## Results

**Figure 1:**
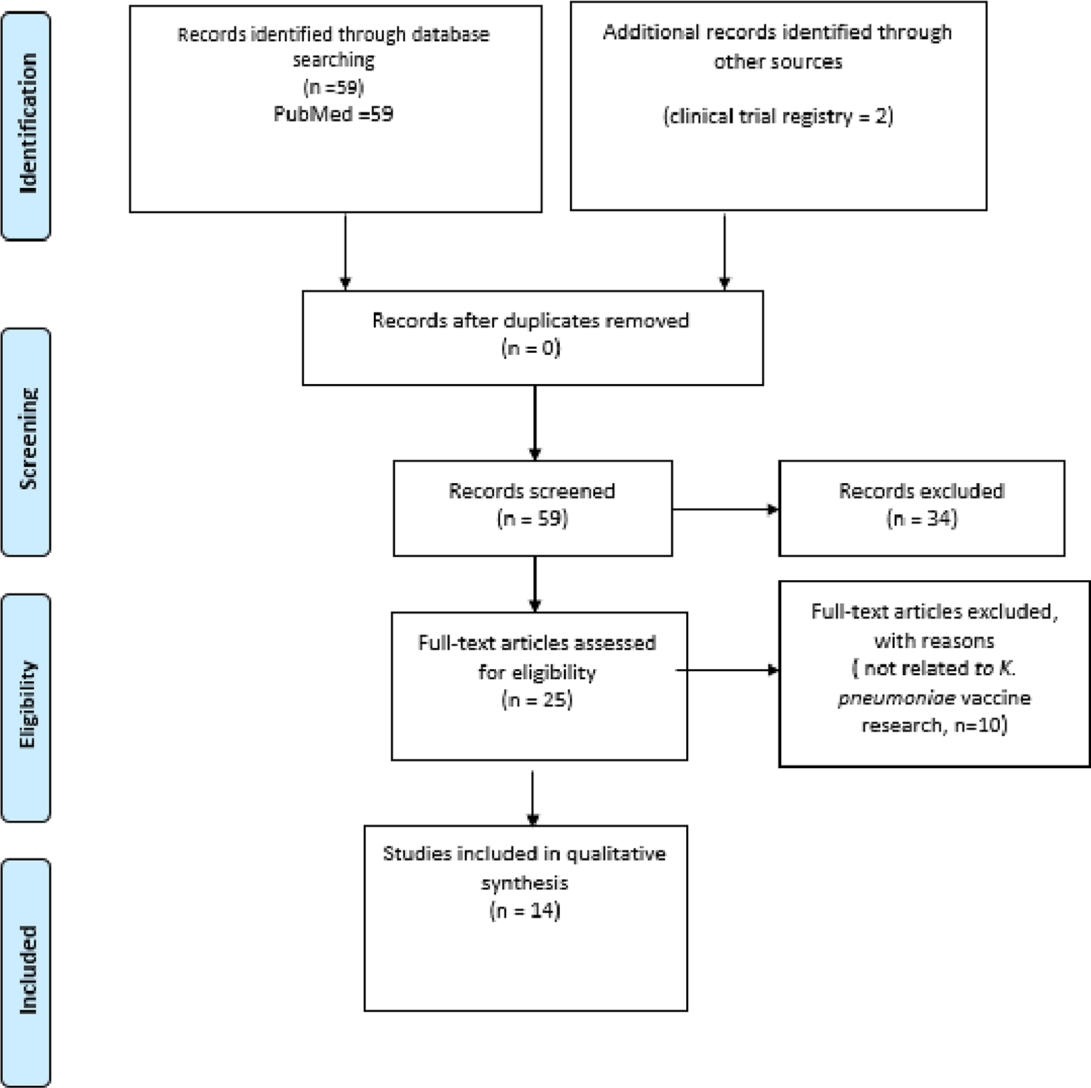
PRISMA flow diagram illustrating the study selection process. *K. pneumoniae, Klebsiella pneumoniae*; PRISMA, Preferred Reporting Items for Systematic Reviews and Meta-Analyses

The review included 14 studies published between 2008 and 2024. Figure 1 shows the process of study selection. Most studies (11/14) were published in the last 5 years (2020–2024).^17^,^1930^ Seven studies were single-country studies conducted in Botswana,^34^ Gambia,^35^ Malawi,^19^ South Africa,^36^ Angola^18^ and two studies from Nigeria.^38 39^ Three studies covered multiple African countries,^17 30 37^ while four others included data from LMICs more broadly.^31^,^3340^

### Disease burden and public health and economic impact of *K. pneumoniae* vaccines in Africa

Surveillance studies have documented the burden of *K. pneumoniae* infections across African countries. One of the most comprehensive pieces of evidence comes from the Child Health and Mortality Prevention Surveillance (CHAMPS) network study,^17^ which found *K. pneumoniae* responsible for 21% (497/2352) of child deaths across six African countries. The burden was particularly high among infants aged 1–11 months (30%) and children aged 12–23 months (28%), with striking geographical variations ranging from 52% in Ethiopia to lower rates in other regions. Another CHAMPS analysis^37^ specifically examining meningitis revealed *K. pneumoniae* as a major pathogen, causing 40.4% (40/99) of hospital-associated and 35.4% (28/79) of community-associated neonatal meningitis deaths. Country-specific studies provide additional evidence. In The Gambia, *K. pneumonia* was identified as the fourth most common pathogen causing invasive bacterial disease, accounting for 5% (12 cases) of cases.^35^ A South African study^36^ showed *K. pneumoniae* causing 13.5% of culture-confirmed bacterial meningitis in infants. In Angola,^18^ found *Klebsiella* species in 30 out of 212 (14.2%) bacterial isolates from infant meningitis cases. Nigerian studies^38 39^ reported *Klebsiella* prevalence of 15.3% in pneumonia blood cultures and 7% in meningitis cases, respectively.

Two studies reported focused on genomic studies that have provided insights into the pathogen’s distribution and characteristics^33^ identified *Klebsiella* as the third most common bacterial pathogen (4.6%, 17/370) in respiratory infections in Madagascar.

### Vaccine and immunisation technology development

Some immunological studies that could support vaccine development have been reported. A study conducted in Botswana demonstrated that lower maternal *Klebsiella* IgG levels were significantly associated with neonatal sepsis (p=0.012), indicating that maternal IgG might be leveraged for broad protection, supporting vaccine feasibility.^34^ Longitudinal genomic surveillance documented temporal trends in resistance and surface antigens, providing valuable data for vaccine development.^30^

Our review found no ongoing or completed clinical trials for *K. pneumoniae* vaccines in African settings.

### Potential research/knowledge gaps

Our review identified several critical gaps in *K. pneumoniae* vaccine research and development in Africa, spanning the three key thematic areas. Current surveillance systems show limited geographical representation across Africa, technical challenges such as poor blood culture sensitivity, hampering accurate disease burden estimation. The absence of immune correlates of protection against *K. pneumoniae* infections presents a gap for vaccine development, while limited clinical trials in African settings is another critical gap as it will be important to determine efficacy in key populations, including pregnant women living with HIV.

Implementation considerations remain crucial, particularly regarding the integration of *K. pneumoniae* vaccination into existing maternal immunisation programmes. A summary of these gaps and our proposed recommendations is presented in table 1, providing a framework for researchers, policymakers and funders to address critical evidence needs in the region.

**Table 1:** Research gaps and strategic recommendations for *K. pneumoniae* vaccine development in Africa

Research gaps and challenges	Recommendations and way forward
Assessment of disease burden and public health and economic impact of vaccines	
Epidemiological surveillance Limited geographical representation in surveillance systems across Africa. Technical limitations in current surveillance, such as poor blood culture sensitivity hinder accurate disease burden estimations.	Strengthen surveillance systems Enhance diagnostic capacity and establish structured surveillance frameworks to capture disease burden, resistance patterns and geographical variability of *K. pneumoniae* infections. Invest in strengthening blood culture capacity across African health facilities to improve diagnostic sensitivity for neonatal sepsis. Need for baseline data collection to assess vaccine impact post-introduction and better characterise at-risk populations. Conduct studies during clinical trials or post-vaccine introductions to evaluate impact of vaccination on AMR. Conduct economic evaluations: Undertake comprehensive cost-effectiveness analyses to strengthen the case for investment in *K. pneumoniae* vaccine research and implementation.
Vaccine development Absence of immune correlates or surrogates of protection against *K. pneumoniae* infections impedes vaccine development and efficacy trials. Few candidates are in early clinical development, and significant technical challenges remain in designing vaccines that target multiple serotypes and resistance profiles effectively. Need for clinical trials in African countries to assess vaccine efficacy and safety, including in vulnerable populations such as neonates and pregnant women living with HIV. Safety assessment when administered together or used in combination with other recommended and near future maternal vaccines (such as influenza, COVID-19, TT, Tdap, RSV and GBS vaccines).	Accelerate vaccine development: Prioritise research on immune correlates of protection. Advance candidates to clinical trials.
Implementation barriers Integration of maternal *K. pneumoniae* vaccination programmes into existing healthcare frameworks, including antenatal care, requires additional research to evaluate feasibility. Strengthening maternal immunisation platforms in Africa and addressing barriers to vaccine access and equity. Gaps in post-introduction surveillance to monitor vaccine safety, effectiveness and broader public health impact.	Expand implementation research: Conduct operational studies to evaluate delivery strategies, healthcare provider engagement and community acceptance of maternal immunisation programmes. Address equity and access: Ensure equitable vaccine distribution by addressing barriers in low-resource settings and targeting high-risk populations. Integrate vaccination into existing platforms: Leverage existing antenatal care and immunisation programmes to facilitate seamless implementation of maternal *K. pneumoniae* vaccination. Promote multisectoral collaboration: Foster partnerships among policymakers, funders, researchers and healthcare providers to align efforts and resources for vaccine development and deployment.

AMR, antimicrobial resistance; GBS, Group B Streptococcus; *K. pneumoniae*, *Klebsiella pneumoniae*; RSV, respiratory syncytial virus; Tdap, tetanus, diphtheria, pertussis; TT, tetanus toxoid.

## Discussion

The development of a maternal vaccine targeting *K. pneumoniae* offers an opportunity to reduce the burden of neonatal sepsis in Africa. Available evidence highlights the substantial impact of *K. pneumoniae* across the continent, particularly in neonatal sepsis, meningitis and mortality. The increasing research attention in recent years has improved understanding of its epidemiology and AMR patterns. However, critical knowledge gaps remain, particularly in surveillance coverage, immunological correlates of protection, and vaccine clinical trials.

Globally, *K. pneumoniae* vaccine development remains at a preclinical to early clinical stage. The most advanced candidate, Kleb4V, a tetravalent bioconjugate vaccine targeting O-antigen polysaccharides of the four most prevalent serotypes (O1v1, O2a, O2afg and O3b), completed a Phase I/II trial in healthy adults aged 18–70 years in Europe, demonstrating acceptable safety and immunogenicity for all four vaccine serotypes.^41^ Other emerging vaccine strategies, including outer membrane vesicle-based platforms and multi-antigen approaches, remain at the preclinical stage.^42 43^ While these global advances are encouraging, the absence of any clinical or preclinical vaccine research activity on the African continent represents a critical gap, given that Africa bears a disproportionate share of the disease burden. Bridging this gap will require deliberate investment in African clinical trial infrastructure and the inclusion of African populations in future efficacy studies.

Public health impact estimates from modelling studies suggest that maternal immunisation against *K. pneumoniae* with 70% efficacy could significantly reduce neonatal sepsis globally. Based on Bayesian analysis of studies involving 2330 neonatal sepsis deaths across 18 predominantly low-middle income countries (2016–2020), a maternal vaccine could prevent approximately 400 000 cases (95% CI 334 523 to 485 442) of neonatal sepsis and 80 000 deaths (95% CI 18 084 to 189 040) annually worldwide.^21^ Given the disproportionately high burden of neonatal sepsis in Africa, the impact in this region is likely to be significant.

Beyond mortality reduction, the economic benefits of a *K. pneumoniae* vaccine extend to alleviating the financial burden on healthcare systems. Neonatal sepsis, particularly from carbapenem-resistant *K. pneumoniae*, results in high hospitalisation costs, prolonged intensive care unit stays and increased medical expenses for families.^21^ A vaccine could contribute to reducing AMR-associated costs, preventing hospital outbreaks and decreasing the need for last-resort antibiotics, which are often expensive and inaccessible in low-resource settings. However, comprehensive economic evaluations in African settings are still lacking and should be prioritised.

Despite documented disease burden, several gaps in epidemiological surveillance hinder accurate estimations of *K. pneumoniae* infections in Africa. The geographic representation of disease burden studies is uneven, with some countries contributing significantly more data than others.

Furthermore, a major challenge in vaccine development is the absence of established immune correlates of protection against *K. pneumoniae* infections. Unlike other maternal vaccines such as tetanus or pertussis, there are no well-defined antibody targets or threshold levels that confer neonatal protection yet. Studies suggest that maternal IgG may play a protective role, with research from Botswana demonstrating a significant association between lower maternal *K. pneumoniae* IgG levels and neonatal sepsis.^34^ However, further immunological studies are needed to inform vaccine design and efficacy evaluation. Additionally, initial vaccine development efforts focused on capsular polysaccharide (CPS) antigens, a major virulence factor. However, the high diversity of *K. pneumoniae* capsular types (over 77 distinct serotypes) has made the development of a broadly protective CPS-based vaccine challenging.^44^ Alternative vaccine strategies have emerged, targeting O polysaccharides, outer membrane proteins and type 3 fimbriae, as well as whole-cell vaccines and outer membrane vesicles.^42 43^ Recent genomic surveillance data have identified key resistance and virulence patterns, which may guide antigen selection for next-generation vaccines.^30^

Integrating a maternal *K. pneumoniae* vaccine into established antenatal care (ANC) immunisation platforms offers a promising strategy, similar to tetanus toxoid. However, potential challenges must be addressed for successful implementation. Strengthening healthcare system preparedness is essential, including investments in cold-chain infrastructure, healthcare provider training and ANC-based vaccine delivery to ensure efficient rollout. Vaccine acceptance and community engagement will also be critical, as maternal vaccines have faced hesitancy.^45 46^ Partnering with community leaders, faith-based organisations and health workers can help build trust and improve uptake.^32 47^ Additionally, co-administration with other maternal vaccines such as influenza, respiratory syncytial virus (RSV) and Group B Streptococcus needs further study to assess immune interactions, safety and efficacy.

Finally, multisectoral collaboration is crucial. Partnerships between global health organisations national policymakers, researchers and funders will be needed to support vaccine development and ensure equitable distribution. Addressing these priorities will be key to advancing a *K. pneumoniae* vaccine and improving neonatal health outcomes in Africa.

The research priorities outlined in this review are broadly consistent with the WHO *K. pneumoniae* Vaccine Value Profile,^21^ which serves as the foundational global reference. However, our review extends these priorities by contextualising them within the African region’s specific realities: limited diagnostic and surveillance infrastructure, the high burden of HIV co-infection among pregnant women and its potential impact on vaccine-elicited immune responses, the implications of high preterm birth rates for vaccine effectiveness and the practical challenges of integrating new maternal vaccines into resource-constrained antenatal care platforms. These region-specific considerations are essential for translating global research priorities into actionable agendas for African researchers, funders, and policymakers.

This review has several limitations that should be acknowledged. First, the search was limited to PubMed as the sole bibliographic database, supplemented by three clinical trial registries (ClinicalTrials.gov, PACTR and ICTRP). While PubMed provides comprehensive indexing of biomedical literature, relevant studies indexed exclusively in other databases. Second, the rapid scoping review approach, while appropriate for mapping an emerging and heterogeneous evidence base, may not capture all available studies. Second, in accordance with established scoping review methodology^22^ and PRISMA-ScR guidelines, formal quality appraisal of included studies was not conducted. This limits the ability to assess the strength of individual study findings but is consistent with the exploratory purpose of scoping reviews. Finally, the research gaps identified were primarily derived from the published literature; a more comprehensive priority-setting exercise would benefit from consultation with regional experts and stakeholders. Despite these limitations, this review has notable strengths. To our knowledge, it is the first scoping review to map *K. pneumoniae* vaccine research specifically in the African context. The use of an analytical framework aligned with the WHO Strategic Framework for Research on Immunisation provides a policy-relevant structure for the synthesis. The inclusion of clinical trial registries broadens the evidence base beyond published literature. The review adheres to established scoping review methodology (JBI) and reporting guidelines (PRISMA-ScR), and identifies actionable, region-specific research priorities.

## Conclusion

A maternal *K. pneumoniae* vaccine has the potential to significantly reduce neonatal sepsis, particularly in resource-limited African settings where disease burden and AMR rates are high. However, critical gaps in epidemiological surveillance, immune correlates and clinical trials must be addressed. Integrating a *K. pneumoniae* vaccine into existing maternal immunisation programmes presents a viable strategy, but healthcare system strengthening, community engagement and economic evaluations are necessary for successful implementation.

## Supplementary material

10.1136/bmjph-2025-004451 online supplemental file 1

## Data Availability

No data are available.
