# Supplementary material for: Klebsiella vaccine research and development in Africa: a rapid scoping review of current evidence and future priorities
Source: BMJ Public Health. 2026 Jul 13;4(Suppl 1):e004451. doi: 10.1136/bmjph-2025-004451 (PMC13365766; doi:10.1136/bmjph-2025-004451)
Supplement: online supplemental file 1 [file bmjph-4-Suppl_1-s001.docx]

**Appendix 1: Search Strategy (PubMed) (01/09/2025)**

| **Search** | **Query** | **Results** |
| --- | --- | --- |
| #4 | Search: #1 AND #2 AND #3 | [59](https://pubmed.ncbi.nlm.nih.gov/?term=%232+AND+%233+AND+%239&sort=date&size=200&ac=no) |
| #3 | Search: (Africa[Mesh] OR Africa*[tiab] OR "Sub Saharan"[tiab] OR "Sub-Saharan"[tiab] OR "North Africa"[tiab] OR "South Africa"[tiab] OR "East Africa"[tiab] OR "West Africa"[tiab] OR "Central Africa"[tiab] OR Angola[tiab] OR Benin[tiab] OR Botswana[tiab] OR "Burkina Faso"[tiab] OR Burundi[tiab] OR "Cabo Verde"[tiab] OR Cameroon[tiab] OR Cameroun[tiab] OR "Canary Islands"[tiab] OR "Cape Verde"[tiab] OR "Central Africa"[tiab] OR "Central African Republic"[tiab] OR Chad[tiab] OR Comoros[tiab] OR Congo[tiab] OR "Cote d'Ivoire"[tiab] OR "Democratic Republic of Congo"[tiab] OR Djibouti[tiab] OR "Eastern Africa"[tiab] OR Eritrea[tiab] OR eSwatini[tiab] OR Ethiopia[tiab] OR Gabon[tiab] OR Gambia[tiab] OR Ghana[tiab] OR Guinea[tiab] OR Guinea-Bissau[tiab] OR "Ivory Coast"[tiab] OR Jamahiriya[tiab] OR Kenya[tiab] OR Lesotho[tiab] OR Liberia[tiab] OR Madagascar[tiab] OR Malawi[tiab] OR Mali[tiab] OR Mauritania[tiab] OR Mauritius[tiab] OR Mayotte[tiab] OR Mozambique[tiab] OR Namibia[tiab] OR Niger[tiab] OR Nigeria[tiab] OR Principe[tiab] OR Reunion[tiab] OR Rwanda[tiab] OR "Sao Tome"[tiab] OR Senegal[tiab] OR Seychelles[tiab] OR "Sierra Leone"[tiab] OR "Saint Helena"[tiab] OR Somalia[tiab] OR "St Helena"[tiab] OR "South Africa"[tiab] OR "Southern Africa"[tiab] OR Sudan[tiab] OR Swaziland[tiab] OR Tanzania[tiab] OR Togo[tiab] OR Uganda[tiab] OR "Western Africa"[tiab] OR "Western Sahara"[tiab] OR Zaire[tiab] OR Zambia[tiab] OR Zimbabwe[tiab]) Sort by: Most Recent | [732,274](https://pubmed.ncbi.nlm.nih.gov/?term=%28Africa%5BMesh%5D+OR+Africa%2A%5Btiab%5D+OR+%22Sub+Saharan%22%5Btiab%5D+OR+%22Sub-Saharan%22%5Btiab%5D+OR+%22North+Africa%22%5Btiab%5D+OR+%22South+Africa%22%5Btiab%5D+OR+%22East+Africa%22%5Btiab%5D+OR+%22West+Africa%22%5Btiab%5D+OR+%22Central+Africa%22%5Btiab%5D+OR+Angola%5Btiab%5D+OR+Benin%5Btiab%5D+OR+Botswana%5Btiab%5D+OR+%22Burkina+Faso%22%5Btiab%5D+OR+Burundi%5Btiab%5D+OR+%22Cabo+Verde%22%5Btiab%5D+OR+Cameroon%5Btiab%5D+OR+Cameroun%5Btiab%5D+OR+%22Canary+Islands%22%5Btiab%5D+OR+%22Cape+Verde%22%5Btiab%5D+OR+%22Central+Africa%22%5Btiab%5D+OR+%22Central+African+Republic%22%5Btiab%5D+OR+Chad%5Btiab%5D+OR+Comoros%5Btiab%5D+OR+Congo%5Btiab%5D+OR+%22Cote+d%27Ivoire%22%5Btiab%5D+OR+%22Democratic+Republic+of+Congo%22%5Btiab%5D+OR+Djibouti%5Btiab%5D+OR+%22Eastern+Africa%22%5Btiab%5D+OR+Eritrea%5Btiab%5D+OR+eSwatini%5Btiab%5D+OR+Ethiopia%5Btiab%5D+OR+Gabon%5Btiab%5D+OR+Gambia%5Btiab%5D+OR+Ghana%5Btiab%5D+OR+Guinea%5Btiab%5D+OR+Guinea-Bissau%5Btiab%5D+OR+%22Ivory+Coast%22%5Btiab%5D+OR+Jamahiriya%5Btiab%5D+OR+Kenya%5Btiab%5D+OR+Lesotho%5Btiab%5D+OR+Liberia%5Btiab%5D+OR+Madagascar%5Btiab%5D+OR+Malawi%5Btiab%5D+OR+Mali%5Btiab%5D+OR+Mauritania%5Btiab%5D+OR+Mauritius%5Btiab%5D+OR+Mayotte%5Btiab%5D+OR+Mozambique%5Btiab%5D+OR+Namibia%5Btiab%5D+OR+Niger%5Btiab%5D+OR+Nigeria%5Btiab%5D+OR+Principe%5Btiab%5D+OR+Reunion%5Btiab%5D+OR+Rwanda%5Btiab%5D+OR+%22Sao+Tome%22%5Btiab%5D+OR+Senegal%5Btiab%5D+OR+Seychelles%5Btiab%5D+OR+%22Sierra+Leone%22%5Btiab%5D+OR+%22Saint+Helena%22%5Btiab%5D+OR+Somalia%5Btiab%5D+OR+%22St+Helena%22%5Btiab%5D+OR+%22South+Africa%22%5Btiab%5D+OR+%22Southern+Africa%22%5Btiab%5D+OR+Sudan%5Btiab%5D+OR+Swaziland%5Btiab%5D+OR+Tanzania%5Btiab%5D+OR+Togo%5Btiab%5D+OR+Uganda%5Btiab%5D+OR+%22Western+Africa%22%5Btiab%5D+OR+%22Western+Sahara%22%5Btiab%5D+OR+Zaire%5Btiab%5D+OR+Zambia%5Btiab%5D+OR+Zimbabwe%5Btiab%5D%29&sort=date&size=200&ac=no) |
| #2 | (Vaccines[Mesh] OR vaccin*[tiab] OR immuniz*[tiab] OR immunis*[tiab] OR "vaccine development"[tiab] OR "vaccine research"[tiab] OR "vaccine design"[tiab]) Sort by: Most Recent | [594,150](https://pubmed.ncbi.nlm.nih.gov/?term=%28Vaccines%5BMesh%5D+OR+vaccin%2A%5Btiab%5D+OR+immuniz%2A%5Btiab%5D+OR+immunis%2A%5Btiab%5D+OR+%22vaccine+development%22%5Btiab%5D+OR+%22vaccine+research%22%5Btiab%5D+OR+%22vaccine+design%22%5Btiab%5D%29&sort=date&size=200&ac=no) |
| #1 | (*Klebsiella*[Mesh] OR *Klebsiella*[tiab] OR "*K. pneumoniae* "[tiab] OR "K pneumoniae"[tiab]) Sort by: Most Recent | [51,311](https://pubmed.ncbi.nlm.nih.gov/?term=%28Klebsiella%5BMesh%5D+OR+Klebsiella%5Btiab%5D+OR+%22K.+pneumoniae%22%5Btiab%5D+OR+%22K+pneumoniae%22%5Btiab%5D%29&sort=date&size=200&ac=no) |
